# Waiting between war and loss: a qualitative study on the experience of ambiguous loss among Syrian refugees

**DOI:** 10.3389/fpsyt.2026.1861927

**Published:** 2026-06-05

**Authors:** Fadim Büşra Keleş, Mustafa Baloglu, Şahin Kesici, Mehmet Ak

**Affiliations:** 1 Department of Guidance and Psychological Counseling, Ministry of National Education, Adana, Türkiye; 2 Department of Special and Gifted Education, College of Education, United Arab Emirates University, Al Ain, United Arab Emirates; 3 Department of Guidance and Psychological Counseling, College of Education, Necmettin Erbakan University, Konya, Türkiye; 4 Department of Mental Health and Psychiatric Disorders, Faculty of Medicine, Necmettin Erbakan University, Konya, Türkiye

**Keywords:** ambiguous loss, forced displacement, grief, refugee mental health, Syrian refugees, war trauma

## Abstract

**Introduction:**

Ambiguous loss refers to individuals' lack of knowledge about the whereabouts or fate of loved ones due to traumatic events such as war or natural disasters. It is associated with significant economic, social, and psychological consequences, including grief, depression, and identity confusion.

**Methods:**

We examined the psychological impact of ambiguous loss among 15 refugees displaced by the Syrian civil war. Participants (22 -53 years old; 9 women, 6 men) had experienced the disappearance or prolonged uncertainty regarding the fate of their spouse, child, sibling, or relative and had lived with uncertainty regarding the fate of their loved one for 4 to 13 years (in some cases, the uncertainty resolved after the interviews). We collected the data through semi-structured interviews and analyzed them using inductive thematic analysis.

**Results:**

Findings revealed emotional oscillation between hope and hopelessness, social isolation, and difficulties adapting to changing roles. Although beliefs and collective rituals served as coping resources, inadequate support and stigma intensified distress.

**Discussion:**

The results indicate that ambiguous loss is embedded in sociocultural contexts and underscore the need for culturally sensitive, family- and community-based psychosocial interventions and supportive national policies.

## Introduction

According to the United Nations High Commissioner for Refugees (1), over 123 million people worldwide are forcibly displaced and compelled to live outside their homelands. Within this context, the civil war in Syria has become the world’s largest source country of refugees (1, 2). More than 4.5 million Syrians are estimated to be living outside the borders of their country (3). It is reported that approximately 3 million Syrians are living in Türkiye (4). The International Organization for Migration’s (IOM) Missing Migrants Project has documented over 75,000 migrant deaths and disappearances worldwide (5). However, information concerning those who have disappeared within Syria, during border crossings, in destination countries, or in refugee camps remains unclear. For example, Tinghög et al. (6) reported that Syrians living in Sweden (64%) reported that family members or loved ones had either died or were missing.

The disappearance of loved ones, accompanied by the absence of information about their fate, is called “*ambiguous loss*” (7). Such disappearances are encountered more commonly during periods of war, political intervention, and forced migration (8). Boss (9) conceptualized ambiguous loss within the framework of stress theory and initially introduced the concept through the case of a father’s ambiguous loss. Subsequently, she expanded the model through her studies with families of soldiers who went missing during the Vietnam and Laos wars. According to Boss (8), ambiguous loss is categorized into two main types. The first type refers to situations in which there is physical absence but continued psychological presence, such as in cases of war, natural disasters, or kidnapping. The second type involves individuals who are physically present but psychologically absent and includes those who have Alzheimer’s disease, alcohol dependence, or emotionally detached (10).

Ambiguous loss has been observed globally in the contexts of war and political violence. In Argentina, the “Mothers of Plaza de Mayo” developed collective rituals to demand information about disappeared relatives (7). Similarly, in Türkiye, the Saturday Mothers and Diyarbakır Mothers have engaged in public actions to clarify the fate of missing loved ones (11). Beyond such organized movements, ambiguous loss also emerges in sudden tragedies, such as the 2014 disappearance of Malaysian Airlines flight MH370 (12), as well as in civil wars, including Sudan (13) and Kosovo (14). Although these examples illustrate the global reach of ambiguous loss, the Syrian refugee context presents unique features due to the prolonged nature of the conflict and post-migration challenges in host countries.

Individuals experience grief in situations such as separation or the loss of loved ones (15); however, in the case of ambiguous loss, because uncertainty prevents closure, individuals are unable to complete the grieving process. Consequently, coping with the loss becomes unbearable (e.g., 15, 16). The effects of ambiguous loss, particularly for immigrants, differ in certain respects (17). For example, immigrants often lose not only loved ones but also their homeland, native language, traditions, and cultural rituals. As a result, such losses are experienced not only cross-sectionally but also intergenerationally, leading to various disruptions (12).

Individuals experiencing ambiguous loss endure a prolonged period of ambiguity with the hope of receiving news from their loved ones. This process may give rise to intense symptoms, such as depression, worry, stress, anxiety, and conflict (18). The persistence of uncertainty can prevent individuals from completing the grieving process and may threaten their psychological integrity. In addition, ambiguous loss creates physiological and behavioral effects. Symptoms such as confusion, headaches, fatigue, gastrointestinal distress, hopelessness, difficulty concentrating, and intense crying may be observed (e.g., 8, 13, 19).

Uncertainty leads to blurred lines in family roles within a social context, a phenomenon described as “boundary ambiguity” and can undermine the individuals’ sense of belonging (7, 8). Unanswered questions, such as who is included in the family system and who should assume roles, may obstruct the pursuit of social support and hinder the collective sharing of grief. Uncertainty may threaten individuals’ psychological integrity by drawing them into a presence-absence paradox (7). Prolonged and intensified uncertainty may trigger disruptions in family structure and endure stress responses (12). Long-term consequences include feelings of guilt, substance abuse, violent tendencies, and identity confusion (20, 21).

During periods of uncertainty, the difficulty individuals face in accessing social support can hinder the collective expression of grief. Societies typically prescribe a limited time frame for mourning (22). After this period, individuals face the risk of exclusion or isolation within their social circles; furthermore, they begin to perceive their emotions as illegitimate (23, 24). Nevertheless, some individuals are more resilient in the face of uncertainty. Such resilience is also related to factors like the duration and intensity of boundary ambiguity (14).

The tragedies associated with global migration movements have also amplified the scale of ambiguous loss. For example, between 2014 and 2018, most of more than 18,000 people who died while attempting to cross the Mediterranean remain unidentified (25), which resulted in tens of thousands of families living in a state of unresolved grief. Despite the high numbers, literature on the experiences of those left behind remains limited (26). This gap is particularly critical given the elevated rates of mental health problems documented among Syrian refugees (27). Limited number of studies, particularly those focusing on individuals who have experienced ambiguous loss in contexts of migration and war, further deepens the theoretical and practical gaps in this field. Syrian refugees often face specific challenges including disappearance of family members at checkpoints, separation from loved ones, and the inability to perform traditional mourning rituals due to the absence of confirmed death. Although ambiguous loss has been studied in various contexts, limited research has examined this phenomenon among Syrian refugees, particularly regarding how war, forced displacement, and post-migration stressors shape the experience of ambiguity and unresolved grief. Specifically, little is known about the emotional, social, and familial dynamics of ambiguous loss among this population. Thus, this study aims to fill this gap by exploring the psychological and social experiences of Syrian refugees living with ambiguous loss, with a focus on their emotional responses, coping mechanisms, and the impact on family dynamics and social relationships. We draw on concepts of cultural trauma (28) and collective memory to frame ambiguous loss not only as an individual psychological experience but also as a socially and culturally mediated phenomenon.

## Methods

### Participants

We used a maximum variation (purposive) sampling strategy to capture diverse perspectives of the participants. We recruited participants from three neighborhoods in Adana (a city bordering Syria) with high concentrations of Syrian residents. The first author visited local community centers and mosques to introduce the study. Initially, 22 individuals were approached; 7 declined to participate (4 men, 3 women). Thus, the final sample included 9 women and 6 men. This gender imbalance reflects that women were more willing to discuss their experiences.

The study sample comprises 15 individuals who have received no information about their loved ones and lacked knowledge regarding their fate. Inclusion criteria required that participants were experiencing ongoing uncertainty regarding the fate of a loved one at the time of data collection. Participants whose loved one’s body was recovered (P1, P2) were included because they had experienced a prolonged period of ambiguous loss prior to the confirmation of death. During interviews, they were asked to reflect on their experiences during that period of uncertainty, not on their bereavement after confirmation. This methodological approach is consistent with previous ambiguous loss research that has included participants whose uncertainty had since resolved, focusing the analysis on the lived experience of the ambiguous period itself (e.g., 29*).*

As shown in **Table 1**, the participants varied in terms of gender, age, duration of loss, and educational level. All participants, aged between 22 and 53 years, were living with their families at the time of the study. Their educational levels ranged from primary education to a college degree. The duration of ambiguous loss experienced by the participants varied from 4 to 13 years and, for some, remained ongoing at the time of the study. Whereas some participants had attended the funerals of their loved ones, a few had witnessed their safe return. The remaining participants, who were still awaiting the return of their loved ones, continued to maintain psychological bonds with them amidst uncertainty. The participants’ experiences were deemed consistent with Boss’s (7) theory of ambiguous loss.

**Table 1:** Participant characteristics.

Participant	Gender	Age	Relationship to the missing person	Duration of ambiguous loss (years)
P1	Man	35	Brother	6 (Body recovered)
P2	Man	27	Cousin	4 (Body recovered)
P3	Man	22	Cousin	13 (Missing)
P4	Woman	48	Son	10 (Missing)
P5	Man	45	Spouse	9 (Missing)
P6	Woman	49	Son	9 (Missing)
P7	Woman	27	Spouse	10 (Missing)
P8	Woman	48	Son	11 (Missing)
P9	Man	52	Spouse	10 (Missing)
P10	Woman	38	Spouse	10 (Missing)
P11	Woman	50	Son	13 (Missing)
P12	Woman	53	Son	11 (Missing)
P13	Woman	29	Father	9 (Found alive)
P14	Woman	43	Son	7 (Found alive)
P15	Man	38	Brother	13 (Found alive)

Participants P13, P14, and P15 were experiencing ongoing ambiguous loss at the time of data collection. Their cases resolved after the interviews, following the political changes in Syria. Participants whose loved one's body was recovered (P1, P2) had also experienced prolonged uncertainty prior to the confirmation of death.

### Interviews

The first author of the study conducted all interviews face-to-face, in Turkish, and at the participants’ dwellings. Participants were proficient in understanding and expressing themselves in Turkish. Care was taken to ensure that the interview environment was quiet and well-ventilated. Interviews lasted from 60 to 120 minutes, and were audio-recorded (1. H 5.E) with the participants’ written and verbal informed consent and transcribed immediately after the interview.

An in-depth, interactive interview approach was utilized, allowing participants to express the meanings they attributed to their experiences in their own words (30). This approach aims to capture how individuals interpret and make sense of their experiences from their own perspectives. The interviewer had experience in qualitative data collection methods and used a semi-structured interview guide during data collection. Priority was given to participants’ narratives, and conversations were allowed to unfold naturally. The interview questions focused on the following areas: the participant’s relationship with the missing person (e.g., “Could you tell us a little about your missing loved one?”); the conditions surrounding the loss (e.g., “Where and how did they disappear? What did you do during this period? How did people around you respond?”); and the individual and social impacts of ambiguous loss (e.g., “How did your roles and responsibilities within the family change during this process?”). All responses were transcribed verbatim and used in their original form for analyses.

Participant quotations cited in the findings were translated into English by the second author, a bilingual-native Turkish speaker. To ensure accuracy, the second author back translated a random selection of 20% of the quotations into Turkish without access to the original versions. The two Turkish versions were compared, and discrepancies were discussed until consensus was reached by all authors. This process confirmed that the translations preserved the original meaning and emotional tone of participants’ statements. We acknowledge that some cultural and emotional nuances may not be fully captured in translation; however, the back-translation procedure minimized this risk.

### Data analysis

The first and second authors coded the interview transcripts manually. During the coding process, we read each transcript in detail to identify meaningful units. Next, they were transformed into explicit codes. Similar codes were clustered into higher-order categories, and these categories were ultimately grouped under themes. The research team discussed coding disagreements openly. Instead of suppressing differences, we used them as analytical resources to deepen our interpretations. Consensus was reached through discussion, but divergent views were documented as well.

We determined data saturation when no new codes, categories, or themes emerged in three consecutive interviews. The sample of 15 participants achieved thematic saturation (31). After the themes emerged from the data, we discussed them in relation to existing literature, including Boss’s (7) theory of ambiguous loss, to identify points of convergence and divergence. We also checked the content consistency and inclusiveness of the themes and merged or divided some themes into categories and subcategories. We organized the themes, categories, and subcategories to conceptualize ambiguous loss and coping styles (32). Although we did not develop any formal codebook, the research team systematically reviewed transcripts, identified codes collaboratively, and grouped them into categories and themes through iterative discussions until we reached complete consensus. Analyses followed the six-phase approach to thematic analysis described by Braun and Clarke (33): Familiarization with the data, generating initial codes, searching for themes, reviewing themes, defining and naming themes, and producing the final report. The first and second authors independently coded the first three transcripts, compared their codes, and resolved discrepancies through discussion. All four authors reviewed the coding framework and contributed to the refinement of themes in regular team meetings. The final thematic structure was reviewed by all authors and checked against the original data to ensure coherence and representativeness. This study adheres to the Consolidated Criteria for Reporting Qualitative Research (COREQ) checklist (34). Whereas coding was inductive, theme interpretation was guided by Boss’s (7) theory of ambiguous loss. This abductive approach allowed us to remain grounded in the data while situating findings within existing theory.

### Researcher positionality and reflexivity

The first author (a Turkish woman) conducted the interviews face-to-face. Although she had previously conducted similar interviews with migrant communities, she did not share the same cultural or linguistic background with the participants. None of the authors spoke Arabic. Participants were given the choice to conduct interviews in Turkish and they felt comfortable expressing themselves in Turkish. However, we acknowledge that Turkish is a second language for all participants, which may have limited the depth of emotional expression, particularly regarding trauma and grief.

To mitigate this risk, the interviewer did not pressure participants regarding time but encouraged them to ask for clarification when they did not understand anything. She explicitly invited them to switch to Arabic if wanted, though none chose to do so. After each interview session, the interviewer summarized the main themes discussed and reviewed them with the participant to confirm accurate understanding. This member-checking process was conducted with all 15 participants, and no discrepancies were identified. Participants’ Turkish proficiency was assessed through self-report and all confirmed comfortable comprehension and expression.

The research team met regularly throughout the data collection process to discuss their own assumptions and potential biases. Disagreements that arose among the researchers during the coding process were resolved through discussions in which each researcher provided a detailed explanation of their interpretations. This process contributed to a more in-depth and reflexive analysis. There were no personal or professional relationships between the researchers and the participants prior to the study.

### Ethical considerations

Scientific Research Ethics Committee of the university where the third and fourth authors were employed officially approved the study before contacting participants [Approval No. 2024/229, dated 01 March 2024]. We provided written informed consent forms and explained them verbally to participants. Participation was voluntary, and confidentiality was protected in line with the principle of anonymity. Given the sensitive nature of trauma, loss, and grief, participants were reminded before each interview that they could pause, skip any question, or withdraw at any time without any consequence. At the interviews, participants were provided with contact information for free psychosocial support services available in their community. A distress protocol was in place: if a participant became visibly distressed, the interviewer would pause the interview, offer a break, and ask whether they wished to continue or stop. No participant required activation of the distress protocol, and none requested early termination of the interview.

### Findings

We organized the findings into five themes and their respective categories: Emotional Confusion (Emotional Oscillation, Waiting with Hope, Survival-Faith); Social Relationships (Supportive-Empathic Approaches, Feeling Misunderstood-Isolated, Social Pressure-Norms, Cognitive-Emotional Contradictions); Expectations (Safety-Future Anxiety, Family Bonds-Missing Members, Realizing the Value of Life); Identity, Roles, and Family Solidarity (Changes in Identity and Roles, Family Solidarity, Changes in Socio-Economic Status); and Blame Dynamics (Internalization, Externalization). These five themes capture distinct dimensions of ambiguous loss: Emotional Confusion (Internal Emotional States), Expectations (Future Orientation), Social Relationships (Interpersonal Dynamics), Identity and Family Roles (Structural Changes), and Blame Dynamics (Attributions of Responsibility). Overlaps between themes reflect the complexity of the phenomenon rather than analytical weakness.

### Emotional confusion

The emotional confusion theme indicates the multifaceted emotional confusion experienced by refugees following the loss of loved ones whose absence could not be confirmed or whose psychological presence could not be fully perceived. These findings match the frozen and incomplete nature of grief described in Boss’s (8) theory of ambiguous loss. **Table 2** presents sample participant statements illustrating this theme.

**Table 2:** Sample participant statements related to the theme of emotional confusion.

Category	Verbatim participant statements
Emotional oscillation	P13 (F-29) *One day, I think he is alive, and the next day I believe he is dead. Living like this is very difficult.* P6 (F–49): *I experience a different emotion every day. Sometimes I feel that he is alive somewhere; other times I feel like I can no longer endure it.* P2 (M–27): *There is always a pain inside me. I cannot fully grieve, nor can I say goodbye to him.* P1 (M–35): *Being trapped in uncertainty, imagining different scenarios every day, and never reaching a conclusion is extremely exhausting for me.* P11 (F–50): *I cried every day and became completely exhausted. I kept thinking to myself, I wish I could at least have seen my son get engaged.*
Waiting with hope	P13 (F–29): *Waiting with hope has destroyed me over time.* P7 (F–27): *I live with the hope of finding him. I am always waiting, thinking that maybe one day he will walk through the door.* P5 (M–45): *I do not want to lose my hope. This hope keeps me standing, but sometimes it also exhausts me deeply.* P2 (M–27): *I made every possible effort to find my cousin and did not want to give up hope.* P9 (M–52): *I am still waiting for her. Until my wife returns, my feet will not leave this place.* P10 (F–38): *Every day, I tell myself that my husband will come back tomorrow.*
Survival-faith	P9 (M–52): *I have to be strong. I must stay standing for my family.* P11 (F–50): *I pray every day, hoping for news. Prayers comfort me a little.* P3 (M–22): *Life is very difficult, but I cannot give up. Maybe one day everything will get better.* P15 (M–38): *I ask God for help, and He is the best guardian.* P6 (F–49): *Everything that happens has a reason. I have accepted this situation and surrendered to it.*

The participants displayed fluctuations between hope and despair, and they mentioned efforts to cope with the difficulties through survival and faith. Emotional uncertainty prolonged the grieving process. The closer the loss within the family circle, the more intense the effect was. Deeper turmoil was experienced in the cases of first-degree kinship loss.

### Social relationships

Syrian refugees’ experiences of ambiguous loss in their social environment were complex and contradictory. We found that supportive and empathetic responses existed together with challenges arising from misunderstanding, loneliness, and social pressures. Participants’ accounts suggest that the social distress caused by the indeterminate nature of the grieving process matches Boss’s (8), (7) conceptualization of disenfranchised grief. **Table 3** presents sample participant statements illustrating this theme.

**Table 3:** Sample participant statements related to the theme of social relationships.

Category	Verbatim participant statements
Supportive–empathic approaches	P1 (M–35): *The support and understanding of some people were very valuable to me.* P3 (M–22): *Our relatives try to support one another as much as they can.* P2 (M–27): *I realized who my true friends were. I began to appreciate more those who never left me alone and were always by my side.* P15 (M–38): *The support of my friends who stood by me and listened to me was a source of comfort.* P11 (F–50): *I felt compassion for the pain that other people were going through.* P2 (M–27): *Some people were indifferent or distant. This disappointed me and sometimes made me angry.*
Feeling misunderstood–isolated	P7 (F–27): *Some people said my husband was fine; others said they had no information about him. There was no one who cared about him as much as I did.* P5 (M–45): *People around me thought I was crazy and left me alone.* P6 (F–49): *His siblings and uncles continued with their lives, but I am still waiting for him in a corner.*
Social pressure–norms	P5 (M–45): *They keep telling me that I should remarry and start a family.* P8 (F–48): *Her husband lost hope; everyone told him to remarry, and he did and continued with his life.* P12 (F–53): *The fact that one of our relatives was going to marry the wife of another relative whose husband had been taken captive—simply because everyone assumed her husband was dead—terrified me.”* P10 (F–38): *I rejected the marriage proposals I received. I believe my husband is alive, but people do not believe this and insist that I should remarry.*
Cognitive–emotional contradictions	P1 (M–35): *I had mixed feelings toward other people.* P15 (M–38): *Sometimes seeing others reunited with their loved ones made me feel jealous, and then I blamed myself for it.* P13 (F–29): *When we started finding bodies, I thought that finding a body was easier than this.* P14 (F–43): *Even when others showed support, I felt pain inside. I felt ungrateful for being treated so well.* P15 (M–38): *Although I sometimes felt empathy for others, I was also struggling to cope with my own pain.* P2 (M–27): *Some people told me to stop hoping and accept reality, while others urged me never to lose hope. Even though I seemed to ignore them, it always made things difficult for me.*

The analyses indicate that women participants—particularly those who experienced the loss of a spouse—were subjected to intense pressure arising from societal norms and traditional expectations, such as pressure to remarry. As illustrated in **Table 3**, participants’ statements about remarriage pressure (e.g., P5, P8, P10, P12) consistently reflected this dynamic. Such social interventions deepen the experience of ambiguous loss and further complicate individuals’ ability to complete the grieving process. By contrast, women who had lost a son reported relatively less social pressure and described the support they received from their social environment as a source of comfort, albeit a partial one. In addition, participants whose relatives’ bodies were recovered stated that they were able to make more effective use of social support mechanisms, as their losses were situated within a more concrete and definable framework. Putting all together, these findings show that social relationships can act both as a source of support and as an additional stressor in the context of ambiguous loss. Participants’ cognitive and emotional conflicts, such as stating that “it would be easier to find the body” or feeling envy toward others’ happiness, show how the internal turmoil created by uncertainty appears in social interactions.

### Expectations

This theme shows how the uncertain state of loss created by war and forced migration deeply changes not only individuals’ current lives but also their perceptions of time, plans, and sense of belonging. The findings suggest that the physical and psychological ambiguity surrounding loss creates deep insecurity and anxiety about the future, causing participants to adopt a way of life centered on “living one day at a time.” Consistent with Pauline Boss’s concept of “frozen grief,” this condition demonstrates a state in which individuals are unable either to return to the past or to move forward into the future. This theme captures participants’ shifting orientations toward the future, including hopes, fears, and meaning-making. **Table 4** presents sample participant statements related to this theme.

**Table 4:** Sample participant statements related to the theme of expectations.

Category	Verbatim participant statements
Safety-future anxiety	P1 (M, 35): *Feeling safe and living a normal life has become much more difficult. My hopes for the future have been replaced by fear.* P2 (M, 27): *Because of conflict and war, I never felt safe where I lived. This destroyed my hopes for the future.* P3 (M, 22): *We try not to get attached here because we fear being sent back from Türkiye. But we also don’t know if Syria will accept us or what will happen to us. In this state of being trapped, we try to survive day by day.* P15 (M, 38): *After my brother’s disappearance, all the balance in our lives collapsed. The dreams we once had for the future were replaced by fear. I know that nothing will ever be the same again.*
Family bonds-missing members	P4 (F, 48): *My God, where is my son? There is no information about him. My son is gone.* P5 (M, 45): *I hoped to find my family, but no one had seen them.* P6 (F, 49): *Since my son disappeared, I am waiting for him every moment.* P7 (F, 27): *After the recent events in Türkiye, my husband and children decided to go to another country. But I will stay here until my husband comes and I see that he is safe.* P8 (F, 48): *My son is gone, and the peace of my home is gone.* P9 (M, 52): *My wife still hasn’t returned. We no longer know where she is.* P12 (F, 53): *My son’s wife and children are slowly getting used to his absence. This is important for them to continue their lives.* P13 (F, 29): *The loss of my father and uncle deeply affected my life and my thoughts about the future. We had nothing but prayer.*
Realizing the value of life	P2 (M, 27): *It made me realize the value of life more deeply. Living in a time full of uncertainty, appreciating every moment and small happiness became important.* P11 (F, 50): *It shows us how deeply war and loss affect our lives.* P14 (F, 43): *After the loss, we try to preserve hope and dreams for the future. I carried this hope for seven years and prayed every day.*

Participants’ statements reveal deep contradictions in how individuals experience family bonds and roles. While waiting for their missing loved ones, women reported that they found it hard to understand how their children and those around them continued with their lives. This finding indicates how ambiguous loss not only has repercussions on the individual but also on the functioning of family systems. On the other hand, some participants saw this traumatic experience as a positive transformation, such as recognizing the value of life and appreciating the significance of small moments. Thus, ambiguous loss can be not only a form of devastation in an individual’s inner world but also a gateway to the search for new meaning.

### Identity, roles, and family solidarity

The theme indicates how chaos in ambiguous loss leads to major changes in personal identities and family roles. In this study, participants were often forced to take on new responsibilities to make up for the absence of missing family members. These role changes required individuals to renegotiate both their personal identities and their positions in the family structure. Sample participant statements illustrating this theme are presented in **Table 5**.

**Table 5:** Sample participant statements regarding the theme of identity, roles, and family solidarity.

Category	Verbatim participant statements
Changes in identity and roles	P15 (M-38): *My son used to be a strong pillar for us. But his disappearance led to questioning—questioning even ourselves: why we existed, why these sufferings found us.*
	P1 (M-35): *Now, I feel like I have to be the father of my brother’s children as well.*
	P6 (F-49): *His siblings and uncles continued with their lives, but I am still waiting for him.*
	P8 (F-48): *When someone disappears from your life, everyone knows that their absence cannot be filled. Yet, in a futile effort, you take on their responsibilities so that their family and loved ones do not feel that absence*.
	P10 (F-38): *We received no news from my husband for years. My family and those around me told me I was young and beautiful and should remarry. But I am still my husband’s wife—how can that be otherwise?*
Family solidarity	
	P1 (M-35): *My mother, my wife, and I are running around for all our relatives’ problems, both financially and emotionally*.
	P3 (M-22): *For example, we do not want women to do many outside tasks. If someone’s husband goes missing and her son is still young, you are obliged to take care of her outside affairs.*
	P7 (F-27): *Everyone tries to help each other, but only to a certain extent. Because a person also has to take care of themselves.*
	P3 (M-22): *I have too many responsibilities on me. You have to be everywhere. This is exhausting for me and for others as well.*
	P10 (F-38): *Together with my children, we imagined my husband’s return and never gave up waiting for him. We tried to continue everything as if he were still present.*
	P13 (F-29): *Because we were all women, we had to go to my uncle’s house; we supported each other, and my uncle took care of us.*
Changes in socio-economic status	
	P6 (F-49): *We left Syria—our home, our reputation, our property, our possessions, our language. We left everything behind.*
	P7 (F-27): *We came to Türkiye because we had to earn money and protect ourselves. I continued waiting; my heart and soul remained in Hama.*
	P11 (F-50): *When we were there, we were well-off people. Even if we were not rich, we lived in good neighborhoods and consumed good-quality products.*
	P15 (M-38): *Here, we have to work extremely hard both financially and emotionally. Our savings and future investments are all gone.*

Syrian refugees mentioned that family solidarity may have functioned both as a support mechanism and as an additional burden. Whereas some received financial and/or emotional support from their family, this support sometimes caused fatigue due to new roles and responsibilities. Women noted taking on new duties that went beyond their traditional roles such as providing for the household and protecting the family’s honor. Therefore, ambiguous loss not only caused individual distress but also led to a structural crisis that affected the entire family system.

### Blame dynamics

Internalization refers to participants’ tendency to attribute responsibility for their loss to themselves (e.g., self-blame, guilt). Externalization refers to attributing responsibility to external factors such as state institutions, security forces, or the political regime. Blame dynamics are about individuals’ efforts to understand traumatic experiences by assigning responsibility and meaning to what has occurred. The findings indicate that participants’ narratives are organized around two primary blame dynamics: internalization and externalization. These processes are closely connected to feelings of guilt and shame, as per the theory of ambiguous loss (8), and show individuals’ ongoing efforts to construct meaning in the face of events that stay largely beyond their control. **Table 6** presents sample participant statements related to the theme of blame dynamics.

**Table 6:** Sample participant statements related to the theme of blame dynamics.

Category	Verbatim participant statements
Internalization	P2 (M-27): *Those who started the war and the conflicts are, of course, guilty, but there is not a single party to blame. We could have realized some things earlier and protected ourselves, but we could not.*
	P7 (F-27): *We cannot say that this or that person is to blame; in the end, the impact of the war on us is clear.*
	P15 (M-38): *I blame myself somewhat for sending my brother out of the city.*
Externalization	P1 (M-35): *States are responsible for the war. Ordinary people suffer from what happens; nothing happens to the rich.*
	P4 (F-48): *The police took my son and said they would interrogate him. After that, nothing. In my view, the police are to blame.*
	P5 (M-45): *At the checkpoint, they questioned my wife and learned that she belonged to a well-known tribe. The system took my wife and said, “We will not release your spouse until the entire clan surrenders.”*
	P8 (F-48): *The tragedy experienced by my son and my family is the result of war and political oppression.*
	P9 (M-52): *My wife disappeared and was subjected to torture. This was done by the state and the security forces.*
	P10 (F-38): *After my husband was taken, I went to all the government offices. I wanted to find some hope, but I was always told, “He is gone now; stop looking, let it go.” Moreover, my husband was taken captive in our home at gunpoint; this is not treatment even criminals receive. It was an arbitrary detention.*
	P11 (F-50): *I state that the regime is responsible for what I experienced. The raids carried out by the regime, the massacre that led to the death of my family, and the arrest of my son—they are behind everything I have lived through.*
	P12 (F-53): *The clashes began because the army entered our village and opened fire everywhere and at everyone.*
	P13 (F-29): *I see the violence and power of the regime behind the events and the losses.*
	P14 (F-43*): The war caused these losses. We did not know where my son was, how he was, or what condition he was in; we could not even find out—they did not tell us, and they threatened us.*

Most participants externalized the experience of loss, attributing responsibility to the war, political authorities, state institutions, and security forces. This externalization is a systemic problem. In contrast, a smaller number of participants internalized the process, blaming themselves, through statements like “We could have questioned their own decisions.” These findings show that blame dynamics are not only individual psychological reactions but also a phenomenon closely linked to the type of loss and the socio-political context.

## Discussion and conclusion

Consistent with Boss’s conceptualization of ambiguous loss (1991, 1999), our findings show that experiences are centered around uncertainty, ongoing grief, and a sense of incompleteness. The subjects of this study were refugees who were forced to relocate due to civil war in Syria. This relocation was not only a physical displacement but also an unidentifiable and unresolvable source of grief (18). They readily reported the psychological burden caused by the inability to obtain definitive information about the fate of missing family members or friends. These findings are similar to the traumas caused by natural disasters such as the 2004 tsunami in Sri Lanka. Individuals who experienced and survived the tsunami and recovered the remains of their loved ones reported experiencing lower levels of prolonged grief and depression compared to those who did not (e.g., 35). Similar findings were reported with people in Bosnia and Herzegovina (36).

As Caruth (37) and LaCapra (38) suggest, traumatic experiences often resist direct representation, leading to narrative fragmentation and a frozen state of waiting—patterns clearly evident in participants’ oscillation between hope and despair and their repeated circular narratives (e.g., P6, P13). Furthermore, Alexander’s (28) concept of cultural trauma highlights that ambiguous losses disrupt not only individual identity but also collective belonging—a disruption reflected in participants’ social isolation and inability to perform shared mourning rituals. Our findings extend Boss’s theory of ambiguous loss in three specific ways. First, by showing how war and political violence create prolonged, state-sponsored uncertainty that differs from natural disasters or accidents. Second, by demonstrating that forced displacement compounds ambiguous loss with the loss of homeland, language, and cultural rituals. Third, by revealing the central role of collective rituals (e.g., communal prayers, almsgiving) and family role restructuring as culturally grounded coping mechanisms among Syrian refugees.

### Emotional confusion, expectations, and meaning-making

Both the findings of this research and the existing literature show that individuals leaving war zones simultaneously carry the trauma of displacement, the psychological burden of ambiguous loss, and the pressure on their new communities. Individuals experiencing ambiguous loss have struggled to accept death due to the void created by the inability to obtain definitive information about the fate of their loved ones, and have continued to wait with hope (14). The fundamental motivation behind this hope is the desire to reunite with the missing person (13). Boss (8) emphasizes that hope is not only an emotional anchor but also functional in terms of creating meaning, maintaining psychological resilience, and activating coping mechanisms. However, Worden (39) draws attention to the dilemma of whether it is healthier to cling to hope or to accept the loss and move on to the grieving process. In some cases, hope can slow progress in the grieving process, making emotional distress chronic (40). It has been found that coping strategies based on hope differ from person to person (16), and spirituality is an important part of this diversity. Religious or cultural values such as patience, waiting, and forgiveness are known to help an individual’s relationship with hope and increase psychological resilience (20, 41). Indeed, many participants stated that they turned to religion or cultural belief systems to make sense of their loss (42). Boss (18) also states that this search for meaning stands at the center of the experience of ambiguous loss.

The findings also carry important implications for clinical and social interventions. Concrete recommendations include ambiguous loss-focused psychoeducation groups, family-based role restructuring, culturally adapted grief counseling integrating religious rituals (e.g., communal prayers, almsgiving), and referral to social services for legal support. In this respect, the study not only supports Boss’s (8), (7) theoretical framework but also points to changes in religious interpretations, ritual practices, family roles, and gender norms. By placing ambiguous loss within individual, cultural, and social dimensions, our study provides an interdisciplinary contribution to the literature.

### Social relationships and disenfranchised grief

Cultural practices and social support systems play a key role in shaping the grieving process. Previous research with Syrian refugees has shown that perceived social support may play a protective role in the relationship between traumatic experiences and post-traumatic growth (43). However, in some societies, cultural norms that limit the time allocated for mourning can create pressure on individuals to complete the process quickly (44, 45). In cases of ambiguous loss, this pressure becomes even more complex. Since death has not been confirmed, individuals cannot perform the established mourning rituals. This leads to the invisibility of grief, incompatibility with social expectations, and isolation (46). As the findings show, participants engage in efforts to create meaning not only individually but also collectively. Strategies for sharing the burden of uncertainty include keeping symbolic objects representing the presence of loved ones at home to keep their memory alive (47); holding communal prayers within the neighborhood; or gathering on specific days to give alms in the name of the deceased. These collective rituals have reduced feelings of loneliness among individuals and enabled the symbolic dimension of grief to be shared (48).

The gendered consequences of ambiguous loss are particularly striking: Women experienced remarriage pressure and expanded domestic responsibilities, whereas men often assumed missing relatives’ breadwinning roles. These patterns suggest that ambiguous loss both reinforces and challenges traditional gender roles, a dynamic that warrants further investigation in refugee settings.

The literature also includes examples where ambiguous losses become visible through public rituals. The “Mothers of Plaza de Mayo” in Latin America offer a striking example (49). This public ritual shows that ambiguous loss is not only an individual experience but also a type of loss that finds its place in social memory and is made visible through cultural rituals. Ambiguous loss includes not only physical absence but also the social, political, and cultural invisibility of death. The vast majority of participants in our study were unable to obtain clear information about the fate of their loved ones, thus disabling both individual grieving processes and environmental support mechanisms. In particular, the inability to access the body prevented the symbolic closure of grief, so that death became unacceptable at both the individual and social levels. In this context, the invisibility of loss and death led the individual to internalize their grief and experience it in isolation from their environment (46, 50). This situation is also related to the concept of disenfranchised grief (51).

The lack of social support during this process not only increases feelings of isolation but also lays the groundwork for stigmatization by the individual’s social environment (23, 51). Hollander (44) emphasizes that such experiences of exclusion weaken individuals’ ties to society and deepen emotional alienation. As vulnerability increases, individuals’ capacity to make meaning of their losses diminishes, and their help-seeking behaviors become increasingly constrained (52). In cases of ambiguous loss, the absence of social rituals leaves individuals struggling with cognitive and emotional confusion throughout the grieving process. Over time, they may even begin to doubt the accuracy of the realities they have constructed (18, 46, 50). This situation leads to frequent oscillations between conflicting emotions such as love, anger, hope, and despair (53). As noted in the literature, participants reported being unable to perform the rituals and traditions specific to the grieving process due to the uncertainty surrounding their losses. Many stated that they did not receive sufficient social support, leading to emotional withdrawal and intense loneliness. However, some participants also emphasized that they experienced empathetic and supportive responses from those around them.

Uncertainty over time leads individuals to question their fundamental beliefs about the reliability, predictability, and meaningfulness of the world (14). The inability to participate in separation rituals prevents a symbolic farewell to the deceased, turning the grieving process into an unresolved and chronic waiting period (54). Such losses influence not only the physical absence but also the ongoing attachment at a relational level (46). Some participants decided to remain where their loved one was last seen, hoping to be there when they returned. While this symbolic loyalty sometimes turns into passive waiting, it becomes a functional part of the coping mechanism for individuals. However, prolonged uncertainty can cause increased suspicion, caution, and emotional withdrawal tendencies toward the environment. This kind of response can harm not only the connection with the past but also the sense of meaning and continuity concerning the future, which can lead to ruptures in fundamental trust (55).

### Identity, roles, and family solidarity

According to Boss (18), ambiguous loss is more than an individual experience; it also reflects a structural problem. Boundary ambiguity describes situations in which family members struggle to define their roles and their relationships with one another, so the limits of belonging and responsibility become unclear. This blurring of family boundaries can delay decision-making and disrupt role functioning, creating additional stress for individuals (56). Family members may also interpret the same loss in different ways, which can lead to conflict within the family, communication problems, and emotional distance (20). In such situations, emotional support within the family becomes an important protective factor, especially when uncertainty continues over time (22). Keeping daily routines and rituals helps preserve a sense of psychological continuity (18). In addition, redefining and flexibly sharing the roles of the missing individual can make adaptation to the loss easier (57).

The findings of this study support the theoretical framework in the literature by revealing the effects of ambiguous loss on family and social roles. It was observed that men tended to take on the responsibilities of missing family members. Although it may appear as a patriarchal cultural practice on the surface, this tendency can be considered an adaptation strategy aimed at preserving the functionality of the family. Similarly, women participants also provided support to their relatives who experienced loss in terms of childcare responsibilities. Indeed, a study conducted after the 9/11 attacks found that children who adopted aunts or uncles as parental figures went through the process more healthily (18). In this context, a flexible and restructuring family structure stands out as an important protective factor in coping with ambiguous loss (45).

The effects of ambiguous loss on family dynamics are not limited only to the psychological level, but also to legal and economic areas. Especially when the missing individual serves as the primary breadwinner in the family, uncertainty brings not only emotional devastation but also economic collapse (58). A study conducted during the civil war in Nepal also reported that relatives of missing persons had significant difficulties getting legal and financial support because they could not obtain death certificates (49).

### Blame dynamics

Individuals experiencing ambiguous loss often report complex emotional reactions, including shame, anger, and guilt, along with damage to their basic sense of trust (15). When these emotions are taken in and internalized, feelings of guilt and helplessness can grow stronger (10, 59). For this reason, externalization—assigning responsibility to outside forces rather than to personal inadequacy—becomes an important part of psychological recovery (7). This process helps build psychological resilience and strengthens individuals’ ability to cope with ongoing uncertainty (60). In line with this framework, most participants in the present study showed a tendency to externalize their experiences. This strategy seemed to help them continue their daily life (24, 61).

The Syrian context differs from other ambiguous loss settings in several ways. First, the prolonged nature of the conflict (over a decade) has created sustained, multigenerational uncertainty. Second, participants frequently attributed disappearances to deliberate state violence (e.g., arrests at checkpoints, torture, arbitrary detention), making ambiguous loss not only a personal tragedy but also a politically inflicted wound. Third, the host country context of Türkiye—with its temporary protection regime and uncertain legal status—adds another layer of ambiguity regarding the future. These features distinguish the Syrian refugee experience from ambiguous loss following natural disasters or short-term conflicts.

In conclusion, as Boss (8) also notes, studies on ambiguous loss become particularly prominent during periods of political violence, war, and forced migration. In this study, the experiences of Syrian participants who went through losses related to war and migration processes show strong parallels with the findings in the literature. The experience of migration includes not only physical displacement but also the loss of elements such as language, tradition, social network, and belonging, thus creating a multi-layered grieving process (13, 22, 62). Therefore, ambiguous loss becomes a multidimensional psychosocial challenge for displaced individuals (63).

While our findings align with existing literature on ambiguous loss in other refugee populations (e.g., 13, 49), the Syrian refugee experience may have unique features. However, a systematic cross-cultural comparison is beyond the scope of this study. Future research should explicitly compare Syrian refugees with other refugee groups to identify context-specific divergences.

In summary, this study demonstrates that ambiguous loss among Syrian refugees is not only an individual grief experience but also a multilayered experience intertwined with family roles, social relationships, cultural rituals, political violence, and post-migration life. The findings highlight the emotional oscillation between hope and despair, the critical role of social support and collective rituals, and the restructuring of family roles as key coping mechanisms. We conclude that psychosocial interventions for this population should be culturally sensitive, family- and community-based, and attentive to the unresolved nature of ambiguous grief.

### Limitations

Although we uncovered important information in this study, it has several limitations. Regarding the sample size, qualitative research typically achieves data saturation with 12–15 participants (31). In this study, saturation was reached with 15 participants, and the data yielded rich, in-depth insights into the experiences of ambiguous loss. Nevertheless, the relatively small sample (*N* = 15) limits the transferability of our findings to other contexts or populations. Although our participants varied in terms of gender, age, and loss characteristics, future studies with larger and more diverse samples across multiple host countries are needed to confirm and extend our results. Therefore, our findings should be interpreted as contextually grounded insights rather than statistically generalizable conclusions.

In addition, as data came from a single geographical region (Türkiye), it may limit the transferability of the results to other contexts. Future research should conduct comparative studies across different host countries, use larger samples, quantitative or mixed-methods, and assess the effectiveness of culturally sensitive intervention programs. This study focused specifically on ambiguous loss experiences and did not systematically examine other important dimensions of the refugee experience, such as prior conflict exposure, sociocultural characteristics of the receiving society, or dynamics of social acceptance and exclusion. Future research should integrate these dimensions into a more comprehensive analytical framework. Despite these limitations, the study contributes to the literature by expanding the theory of ambiguous loss through the experiences of Syrian refugees. This also helps broaden its cultural and social dimensions. In particular, by emphasizing culturally specific coping strategies—religious beliefs, collective rituals, and the redefinition of family roles—that support the conceptualization of ambiguous loss as an individual phenomenon and also as a social and structural process. This original contribution brings an interdisciplinary perspective to the existing literature and offers a basis for developing actionable recommendations for psychosocial support programs for migrant communities. As noted in the reflexivity section, conducting interviews in Turkish, a second language for all participants, may have limited the depth and nuance of emotional expression, particularly regarding trauma and grief.

## Data Availability

The datasets presented in this article are not readily available because the research data supporting the conclusions of this article are not publicly available due to their sensitive nature (qualitative interviews with trauma-affected refugees discussing war, disappearance, and political violence). Anonymized data may be made available by the authors upon reasonable request, subject to ethical and confidentiality restrictions. Requests to access the datasets should be directed to Fadim Büşra Keleş¸ fbusrak93@gmail.com.

## References

[1] United Nations High Commissioner for Refugees [UNHCR] . Global trends: Forced displacement in 2024 (2025). Available online at: https://www.unhcr.org/global-trends (Accessed June 1, 2025).

[2] United Nations International Children’s Emergency Fund [UNICEF] . Report on analysis for out-of-school Syrian children (2021). Available online at: https://www.unicef.org/turkiye/media/18036/file/SUR%C4%B0YEL%C4%B0%20OKUL%20DI%C5%9EI%20KALMI%C5%9E%20%C3%87OCUKLAR%20%C4%B0LE%20%C4%B0LG%C4%B0L%C4%B0%20ANAL%C4%B0Z%20RAPORU.pdf (Accessed June 1, 2025).

[3] UNICEF, UNHCR, & WFP . Returning home is a process, not a moment: UN agencies pledge stronger joint action to support Syrian refugees (2026). Available online at: https://www.wfp.org/news/returning-home-process-not-moment-un-agencies-pledge-stronger-joint-action-support-Syrian (Accessed June 1, 2025).

[4] Republic of Turkey, Ministry of Interior, Directorate General of Migration Management . Temporary protection (2025). Available online at: https://en.goc.gov.tr/temporary-protection (Accessed June 1, 2025).

[5] International Organization for Migration . Missing migrants project: Global deaths and disappearances. International Organization for Migration (2026). Available online at: https://missingmigrants.iom.int (Accessed June 1, 2025).

[6] Tinghög P Malm A Arwidson C Sigvardsdotter E Lundin A Saboonchi F . Prevalence of mental ill health, traumas, and postmigration stress among refugees from Syria resettled in Sweden after 2011: A population-based survey. BMJ Open. (2017) 7:e018899. doi: 10.1136/bmjopen-2017-018899. PMID: 29289940 PMC5778338

[7] Boss P . Loss, trauma, and resilience: Therapeutic work with ambiguous loss. New York, NY, USA: W. W. Norton & Co (2006).

[8] Boss P . Ambiguous loss: Living with frozen grief. Harvard Ment Health Letter. (1999) 16:4–6. 10521908

[9] Boss P . The role of intuition in family research: Three issues of ethics. Contemp Family Ther. (1987) 9:146–59. doi: 10.1007/BF00890270. PMID: 30311153

[10] Betz G Thorngren JM . Ambiguous loss and the family grieving process. Family J. (2006) 14:359–65. doi: 10.1177/1066480706290052

[11] Alıcı N Falschebner P . Navigating non-transition: Grassroots victim–survivor mobilization and the pursuit of transitional justice in Turkey and Morocco. J Hum Rights Pract. (2025) 17:675–96. doi: 10.1093/jhuman/huaf012

[12] Boss P . The context and process of theory development: The story of ambiguous loss. J Family Theory Rev. (2016) 8:269–86. doi: 10.1111/jftr.12152. PMID: 40046247

[13] Luster T Qin D Bates L Johnson D Rana M . The lost boys of Sudan: Coping with ambiguous loss and separation from parents. Am J Orthopsychiatry. (2009) 79:203–11. doi: 10.1037/a0015559. PMID: 19485637

[14] Boss P . Ambiguous loss in families of the missing. Lancet. (2002) 360:39–40. doi: 10.1016/s0140-6736(02)11815-0. PMID: 12504498

[15] Testoni I Franco C Palazzo L Iacona E Zamperini A Wieser MA . The endless grief in waiting: A qualitative study of the relationship between ambiguous loss and anticipatory mourning amongst the relatives of missing persons in Italy. Behav Sci. (2020) 10:110. doi: 10.3390/bs10070110. PMID: 32640528 PMC7408511

[16] Boss P . The trauma and complicated grief of ambiguous loss. Pastoral Psychol. (2010) 59:137–45. doi: 10.1007/s11089-009-0264-0. PMID: 30311153

[17] Falicov CJ . Ambiguous loss: Risk and resilience in Latino immigrant families. In: Suárez-Orozco MM Suárez-Orozco C Baolian Qin D , editors.The new immigration: An interdisciplinary reader. New York, NY, USA: Taylor & Francis (2005). p. 197–206.

[18] Boss P . Ambiguous loss research, theory, and practice: Reflections after 9/11. J Marriage Family. (2004) 66:551–66. doi: 10.1111/j.0022-2445.2004.00037.x. PMID: 40046247

[19] Rosen LN Teitelbaum JM Westhuis DJ . Children's reactions to the Desert Storm deployment: Initial findings from a survey of Army families. Military Med. (1993) 158:465–9. doi: 10.1093/milmed/158.7.465 8351048

[20] Boss P Yeats JR . Ambiguous loss: A complicated type of grief when loved ones disappear. Bereavement Care. (2014) 33:63–9. doi: 10.1080/02682621.2014.933573. PMID: 37339054

[21] Sobel S Cowan CB . Ambiguous loss and disenfranchised grief: The impact of DNA predictive testing on the family as a system. Family Process. (2003) 42:47–57. doi: 10.1111/j.1545-5300.2003.00047.x. PMID: 12698598

[22] Solheim C Zaid S Ballard J . Ambiguous loss experienced by transnational Mexican immigrant families. Family Process. (2016) 55:338–53. doi: 10.1111/famp.12130. PMID: 25619113

[23] Doka KJ . Disenfranchised grief: New directions, challenges and strategies for practice. Champaign, IL, USA: Research Press (2002).

[24] Rycroft F Perlesz A . Speaking the unspeakable: Reclaiming grief and loss in family life. Aust New Z J Family Ther. (2001) 22:57–65. doi: 10.1002/j.1467-8438.2001.tb01310.x. PMID: 41531421

[25] Mirto G Robins S Horsti K Prickett PJ Ruiz Verduzco D Toom V . Mourning missing migrants: Ambiguous loss and the grief of strangers. In: Cuttitta P Last T , editors.Border deaths: Causes, dynamics and consequences of migration-related mortality. Amsterdam, Netherlands: Amsterdam University Press (2019). p. 103–16.

[26] Wayland S Maple M McKay K Glassock G . Holding on to hope: A review of the literature exploring missing persons, hope and ambiguous loss. Death Stud. (2016) 40:54–60. doi: 10.1080/07481187.2015.1068245. PMID: 26207394

[27] Aysazci-Cakar F Schroder T Hunt N . A systematic review of prevalence and correlates of post-traumatic stress disorder, depression and anxiety in displaced Syrian population. J Affect Disord Rep. (2022) 10:100397. doi: 10.1016/j.jadr.2022.100397. PMID: 38826717

[28] Alexander JC . Toward a theory of cultural trauma. In: Alexander JC Eyerman R Giesen B Smelser NJ Sztompka P , editors.Cultural trauma and collective identity. Berkeley, CA, USA: University of California Press (2004). p. 1–30.

[29] Danon A Dekel R Horesh D . Between mourning and hope: A mixed-methods study of ambiguous loss and posttraumatic stress symptoms among partners of Israel defence force veterans. psychol Trauma: Theory Research Practice Policy. (2024) 17:795–804. doi: 10.1037/tra0001794. PMID: . Advance online publication. 39347732

[30] Holstein JA Gubrium JF . The active interview. Thousand Oaks, CA, USA: Sage (1995).

[31] Guest G Bunce A Johnson L . How many interviews are enough? An experiment with data saturation and variability. Field Methods. (2006) 18:59–82. doi: 10.1177/1525822X05279903

[32] Alhazmi AA Kaufmann A . Phenomenological qualitative methods applied to the analysis of cross-cultural experience in novel educational social contexts. Front Psychol. (2022) 13:785134. doi: 10.3389/fpsyg.2022.785134. PMID: 35548502 PMC9082033

[33] Braun V Clarke V . Using thematic analysis in psychology. Qual Res Psychol. (2006) 3:77–101. doi: 10.1191/1478088706qp063oa

[34] Tong A Sainsbury P Craig J . Consolidated criteria for reporting qualitative research (COREQ): a 32-item checklist for interviews and focus groups. Int J For Qual Health Care. (2007) 19:349–57. doi: 10.1093/intqhc/mzm042. PMID: 17872937

[35] Isuru A Hewage SN Bandumithra P Williams SS . Unconfirmed death as a predictor of psychological morbidity in family members of disappeared persons. Psychol Med. (2019) 49:2764–71. doi: 10.1017/S0033291718003793. PMID: 30585557

[36] Powell S Butollo W Hagl M . Missing or killed: The differential effect on mental health in women in Bosnia and Herzegovina of the confirmed or unconfirmed loss of their husbands. Eur Psychol. (2010) 15:185–92. doi: 10.1027/1016-9040/a000018

[37] Caruth C . Unclaimed experience: Trauma, narrative, and history. Baltimore, MD, USA: Johns Hopkins University Press (1996).

[38] LaCapra D . Writing history, writing trauma. Baltimore, MD, USA: Johns Hopkins University Press (2001).

[39] Worden JW . Grief counseling and grief therapy: A handbook for the mental health practitioner. 4th ed. New York, NY, USA: Springer (2009).

[40] Barakovic D Avdibegovic E Sinanovic O . Depression, anxiety and somatization in women with war missing family members. Materia Socio-Medica. (2013) 25:199–202. doi: 10.5455/msm.2013.25.199-202. PMID: 24167436 PMC3804435

[41] Pargament KI . The psychology of religion and coping: Theory, research, practice. New York, NY, USA: Guilford Press (1997).

[42] Goodman J . Coping with trauma and hardship among unaccompanied refugee youths from Sudan. Qual Health Res. (2004) 14:1177–96. doi: 10.1177/1049732304265923. PMID: 15448294

[43] Altınışık MS Şanlı E . The moderating role of perceived social support in the relationship between the impact of events and post-traumatic growth among Syrian refugees. Societies. (2024) 14:107. doi: 10.3390/soc14070107. PMID: 30654563

[44] Hollander T . Ambiguous loss and complicated grief: Understanding the grief of parents of the disappeared in Northern Uganda. J Family Theory Rev. (2016) 8:294–307. doi: 10.1111/jftr.12153. PMID: 40046247

[45] Boss P . The myth of closure: Ambiguous loss in a time of pandemic and change. New York, NY, USA: W. W. Norton & Company (2021).

[46] Boss P . Ambiguous loss theory: Challenges for scholars and practitioners. Family Relations. (2007) 56:105–10. doi: 10.1111/j.1741-3729.2007.00444.x. PMID: 40046247

[47] Hirschberger G . Collective trauma and the social construction of meaning. Front Psychol. (2018) 9:1441. doi: 10.3389/fpsyg.2018.01441. PMID: 30147669 PMC6095989

[48] O’Rourke T Spitzberg BH Hannawa AF . Grief rituals: Aspects that facilitate adjustment to bereavement. OMEGA—Journal Death Dying. (2011) 63:119–37. doi: 10.2190/OM.63.2.b. PMID: 21842662

[49] Robins S . Ambiguous loss in a non-western context: Families of the disappeared in post-conflict Nepal. Family Relations. (2010) 59:253–68. doi: 10.1111/j.1741-3729.2010.00600.x. PMID: 40046247

[50] Rosenblatt PC . Recovery following bereavement: Metaphor, phenomenology, and culture. Death Stud. (2008) 32:6–16. doi: 10.1080/07481180701741228. PMID: 18646394

[51] Wayland S Maple M . ‘An all-consuming cumulonimbus of pain:’ A scoping review exploring the impact of ambiguous loss when someone is missing and the counselling interventions relevant to the experience. Bereavement Care. (2020) 39:21–9. doi: 10.1080/02682621.2020.1728089. PMID: 37339054

[52] Werner-Lin A Moro T . Unacknowledged and stigmatized losses. In: Walsh F McGoldrick M , editors.Living beyond loss: Death in the family. New York, NY, USA: W. W. Norton & Company (2004). p. 247–71.

[53] Klass D . Continuing conversation about continuing bonds. Death Stud. (2006) 30:843–58. doi: 10.1080/07481180600886603. PMID: 17004368

[54] Özgeldi EB Gölge ZB . Ambiguous loss: Learning to live with ambiguity. Turkish psychol Articles. (2018) 21:108–20. doi: 10.31828/tpy1301996120180618m000001

[55] Herman JL . Trauma and recovery: The aftermath of violence-from domestic abuse to political terror. 2nd ed. New York, NY, USA: Basic Books (2015).

[56] Pincus S House R Christenson J Adler L . The emotional cycle of deployment: A military family perspective. US Army Med Department J. (2001) 4:15–23.

[57] Boss P Couden BA . Ambiguous loss from chronic physical illness: Clinical interventions with individuals, couples, and families. J Clin Psychol. (2002) 58:1351–60. doi: 10.1002/jclp.10083. PMID: 12412146

[58] Blaauw M Lähteenmäki V . Denial and silence or acknowledgement and disclosure. Int Rev Red Cross. (2002) 84:767–83.

[59] Boss PG Greenberg JR Pearce-McCall D . Measurement of boundary ambiguity in families. St. Paul, MN, USA: Minnesota Agricultural Experiment Station (1990). Available online at: https://conservancy.umn.edu/server/api/core/bitstreams/75accd96-85ba-4ebf-8f46-cc50f78f27f0/content (Accessed June 1, 2025). Report No. 593.

[60] Bonanno GA . Loss, trauma, and human resilience. Am Psychol. (2004) 59:20–8. doi: 10.1037/0003-066X.59.1.20. PMID: 14736317

[61] Silove D . The ADAPT model: A conceptual framework for mental health and psychosocial programming in post conflict settings. Intervention. (2013) 11:237–48. doi: 10.1097/WTF.0000000000000005. PMID: 42050966

[62] Miller KE Rasmussen A . The mental health of civilians displaced by armed conflict: An ecological model of refugee distress. Epidemiol Psychiatr Sci. (2017) 26:129–38. doi: 10.1017/S2045796016000172. PMID: 27040595 PMC6998767

[63] Perez RM . Lifelong ambiguous loss: The case of Cuban American exiles. J Family Theory Rev. (2016) 8:324–40. doi: 10.1111/jftr.12147. PMID: 40046247

